# Ablation of miR-144 increases vimentin expression and atherosclerotic plaque formation

**DOI:** 10.1038/s41598-020-63335-7

**Published:** 2020-04-09

**Authors:** Quan He, Fangfei Wang, Takashi Honda, Kenneth D. Greis, Andrew N. Redington

**Affiliations:** 1 The Heart Institute, Cincinnati Children’s Hospital Medical Center, Cincinnati, Ohio, United States of America; 2 College of Medicine, University of Cincinnati, Cincinnati, Ohio, United States of America

**Keywords:** Cardiovascular biology, Experimental models of disease

## Abstract

It has been suggested that miR-144 is pro-atherosclerotic via effects on reverse cholesterol transportation targeting the ATP binding cassette protein. This study used proteomic analysis to identify additional cardiovascular targets of miR-144, and subsequently examined the role of a newly identified regulator of atherosclerotic burden in miR-144 knockout mice receiving a high fat diet. To identify affected secretory proteins, miR-144 treated endothelial cell culture medium was subjected to proteomic analysis including two-dimensional gel separation, trypsin digestion, and nanospray liquid chromatography coupled to tandem mass spectrometry. We identified 5 gel spots representing 19 proteins that changed consistently across the biological replicates. One of these spots, was identified as vimentin. Atherosclerosis was induced in miR-144 knockout mice by high fat diet and vascular lesions were quantified by Oil Red-O staining of the serial sectioned aortic root and from en-face views of the aortic tree. Unexpectedly, high fat diet induced extensive atherosclerosis in miR-144 knockout mice and was accompanied by severe fatty liver disease compared with wild type littermates. Vimentin levels were reduced by miR-144 and increased by antagomiR-144 in cultured cardiac endothelial cells. Compared with wild type, ablation of the miR-144/451 cluster increased plasma vimentin, while vimentin levels were decreased in control mice injected with synthetic miR-144. Furthermore, increased vimentin expression was prominent in the commissural regions of the aortic root which are highly susceptible to atherosclerotic plaque formation. We conclude that miR-144 maybe a potential regulator of the development of atherosclerosis via changes in vimentin signaling.

## Introduction

Atherosclerotic cardiovascular diseases including ischemic heart disease and cerebrovascular disease (mainly ischemic stroke) are the leading cause of death in the world^1^. Atherosclerosis is a chronic arterial disease complexed with inflammatory disease and metabolic syndrome. It is characterized by buildup of fibro-fatty plaques in the arterial walls. Atherosclerotic plaques contain fat, fibrotic proteins, and cells including endothelial cells, vascular smooth muscle cells, macrophages, and platelets.

Although its exact pathogenesis remains to be fully elucidated, initiation of atherosclerosis is associated with endothelial dysfunction and intimal damage, followed by recruitment of monocytes from circulation to the artery wall attracted by inflammatory mediators and endothelial cell adhesion molecules. Vascular smooth muscle cells also migrate towards the intima, some transdifferentiating into macrophage-like foam cells after lipid-loading from circulating lipoproteins, especially low density lipoprotein (LDL)’s^2^, and some forming a fibrous plaque cap. Besides macrophages, Allahverdian *et al*. report that about 50% foam cells are derived from vascular smooth muscle cells^3^. Platelets also play a key role in atherogenesis and its consequences. Platelets attach to endothelial cells via glycoprotein Ib and one of the many resulting platelet-endothelial interactions is increased endothelial-associated von Willebrand factor (VWF), particularly in a multimerized form, which interacts with platelet glycoproteins and integrins^4^. The A1 domain of VWF contains binding sites for platelet glycoprotein Ib, heparin, collagen, and ristocetin; and its A2 domain contains a binding site for vimentin^5^. Vimentin, a widely expressed intermediate filament protein, is essential for the formation of VWF strings on endothelial cells, trapping platelets, and resulting in initiation of atherosclerosis^4–6^. Vimentin is therefore a potential regulator of atherogenesis, and a potential therapeutic target.

MicroRNA’s (miRNAs) are short, single strands of RNA composed of 18–24 nucleotides. Their predominant function is as negative regulators of protein synthesis via binding to specific sites in the 3’ untranslated region of the target mRNA, inhibiting its translation. They have widespread regulatory functions in multiple diseases, including atherosclerosis. miRNAs involved in altering bioprocesses of endothelial cells, vascular smooth muscle cells, and monocytes/macrophages, or interfering cholesterol homeostasis will be expected pro- or anti-atherosclerosis^7,8^. For example, miR-144 (transcribed with miR-451 in cluster) has been suggested to be pro-atherosclerotic by targeting ATP binding cassette protein1 (ABCA1) resulting in interrupted cholesterol metabolism, and in a study performed in ApoE KO mice administered high fat diet (HFD), an agomir (a type of specially labeled and chemically modified double strand miRNA) of miR-144-3p accelerated plaque formation through impairing reverse cholesterol transport and promoting pro-inflammatory cytokine production ^9–11^. These data, while interesting, do not allow a complete understanding of the role of miR-144 in atherogenesis. Though many miR-144 targets have been individually confirmed, the majority of the approximately 1000 targets predicted by TargetScan have not been confirmed, and loss of miR-144 function has not been examined. In this study, we took advantage of powerful nanospray liquid chromatography coupled to tandem mass spectrometry proteomics, identified vimentin as a target of miR-144, and employed primary cultured cardiac endothelial cell, miR-144/451 KO fed HFD, and mice injected with synthetized miR-144 as model systems to examine the impact of loss of miR-144 on vimentin expression and atherogenesis.

## Material and methods

### Materials

miR-144, antigomiR-144, and scrambled control miRNA as described previously^12^ were synthetized by Eurofins Genomics AT GmbH (Vienna, Austria). To increase their stability, these microRNAs were modified with 3’-cholesterol, 3 x phosphorothioate 3’-side, 2 x phosphorothioate 5’-side, and 2’-O-methyl-RNA. Rabbit monoclonal anti-vimentin antibody was ordered from Abcam, rabbit polyclonal anti-ABCA1 antibody was from Novus Biologicals. HRP-conjugated goat anti-rabbit IgG (H + L) sencondary antibody was from Thermo Fisher and Texas Red-conjugated goat anti-rabbit IgG was from Vector Laboratories. Antibody dilution is followed as manufactory’s suggestions.

### Animal experiment

Animal care and experimental protocols were approved by the Institutional Animal Care and Use Committees of the Cincinnati Children’s Hospital Medical Center in accordance with the Animal Welfare Act (AWA) and PHS Policy on Humane Care and Use of Laboratory Animals (Protocol Number: 2018-0026). Mice were housed in a fully equipped animal facility, with free access to food and water ad libitum, and on a 12 h dark/light cycle. miR-144/451 knockout (KO) mice were provided by Dr. Mitchell Weiss from St. Jude Children’s Research Hospital (Memphis, TN), bred in our animal facility, and gene typed as described previously^13^. For vimentin characterization, 9-week old male miR-144 KO mice and wild type (WT) littermates were sacrificed, and blood and heart were harvested for vimentin assays. For miR-144 injection, 8-week male C57BL/6 mice ordered from Charles River (Mattawan, MI) were injected with 40 mg/kg miR-144 via tail vein as we have previously described^12^ and repeated for 3 times, every 4 weeks. The mice were sacrificed 2 weeks after the last miR-144 injection. Blood and heart tissue were collected for vimentin assays. To induce atherosclerosis, miR-144 KO mice and WT littermates were fed HFD (D12109Ci, Research Diet Inc, New Brunswick, NJ), after weaning, for 48 weeks.

### Cell culture experiment

Primary cardiac endothelial cells, isolated from mouse heart, were purchased from Cell Biologics (Chicago, IL). The cells were cultured on a gelatin-coated plate and expanded according to the manufacturer’s directions. Passage 4 cells were used for experiments. The cells were incubated with miR-144 or transfected by Lipofectamine RNAiMAX from Thermo Fisher Scientific (Waltham, MA) for 48 h after overnight serum starvation. The culture medium and cells were harvested for protein or RNA analysis.

### Proteomics

The endothelial cells were plated on a 100 mm culture plate overnight, serum-free for 24 h, and incubated with miRNA for 48 h. The culture medium was collected and dead cells were cleared by centrifugation. The culture medium was concentrated by Sartorius (Bohemia, NY) centrifugal concentrator with a MWCO of 3,000 kD. The samples were buffer exchanged to GE DeStreak rehydration buffer by centrifuging through Amicon 3 kD filters. Protein concentration was determined with a protein quantification kit from Pierce Biotechnology (Waltham, MA), and BSA was used as standard. Samples (20 µg) were separated on duplicated two-dimensional gels and subsequently silver stained. The gels were scanned and imported into The Nonlinear Dynamics SameSpots program for image analysis. The spots that had a power value of 0.8 or greater were chosen for further analysis. The spots were excised from multiple gels across all groups, reduced with dithiothreitol, alkylated with Iodoacetamide, and digested with trypsin as described previously^14^. The peptides were extracted, dried in a speed vac, and re-suspended in 7 µl of 0.1% formic acid. Five microliters of each sample was analyzed by nanospray liquid chromatography coupled to tandem mass spectrometry and searched against the Musculus database using the ProteinPilot program (Sciex), as previously described^14^.

### Real-time PCR

Cultured cardiac endothelial cells were harvested with Trizol Reagent from Thermo Fisher Scientific (Waltham, MA) and total RNA was isolated following the manufacturer’s direction. Vimentin mRNA and miR-144 levels were determined by quantitative reverse transcription polymerase chain reaction using TaqMan Gene Expression Assays and TaqMan microRNA Assays kits from Invitrogen (Carlsbad, CA) according to manufacturer’s instructions respectively. The relative levels of vimentin mRNA and miR-144 were determined using the ΔΔCt method^15^ and RNA U6 or 18 s rRNA were used to normalize.

### Western blot

Western blotting was performed as previously described^16^. For cell culture medium samples, the proteins from 500 µl culture medium was precipitated with methanol and chloroform. The precipitated proteins were re-solubilized with laminal sample buffer and boiled for 5 min before undergoing acrylamide gel electrophoresis. For plasma samples, 20 µg of total protein was used for this analysis. As we lacked house-keeping proteins in the culture medium and plasma, a major protein band from MemCode Reversible Protein Stain Kit (Pierce Biotechnology) for polyvinylidene difluoride membrane was used for loading control. The Western blot bands were corrected for the major protein band after quantification by Image J and expressed as a ratio of western blot band and major protein band.

### Plasma cholesterol assay

Plasma was obtained via centrifugation of blood at 1,000 g for 15 min at 4 °C using heparin as an anticoagulant. Fresh plasma (50 µl) was used to separate high density lipoprotein from LDL/very low-density lipoprotein (VLDL) for cholesterol assays with a kit from BioVision (Milpitas, CA) following the kit direction. Remaining plasma was aliquoted and stored at −80 °C for ELISA and Western blot assays.

### ELISA

Plasma vimentin levels were determined using a mouse vimentin ELISA kit from LifeSpan BioSciences (Seattle, WA) following its user manual.

### Heart assessment

Cardiac function was noninvasively evaluated by echocardiography as described previously^16^ before termination of the animal experiment.

### Histology

By end of the experiment, mice were sacrificed by overdose of pentobarbital. The animal was *in situ* fixed by perfusion of formalin for 15 min and re-flashed with PBS. The heart and aortic tree were isolated together, and the aorta subsequently separated in the middle of ascending aorta between aortic root and innominate artery. The aortic tree was subjected to en-face Oil red-O staining for atherosclerosis analysis after removal of exterior fat. The heart was weighed for heart weight/body weight ratio. The aortic root was then dissected from the ventricle and embedded in OCT for atherosclerotic plaque quantification. Right ventricular dilatation was evaluated based on images of the cross sliced heart, quantified using Image J software, and expressed as the perimeter ratio of right ventricle and the whole heart. Heart slices were preserved in OCT for histological analysis. The liver was also harvested, weighed, and preserved in OCT for fatty liver analysis.

### Atherosclerosis assessment

Atherosclerosis burden was evaluated by the serial sectioned aortic root and en-face view of the aortic tree after Oil Red-O staining. Quantification was performed using Image J image-analysis software.

### Liver assessment

Adjacent frozen sections of liver were subjected to both hematoxylin and eosin, and Oil Red-O stains. The lipids droplets in the liver were apparent as round empty spaces left behind when the lipids droplets were removed by xylene during tissue processing for H&E stain, and red dots for Oil Red-O stain respectively. The severity of fatty liver was quantified from the area of oil droplets identified with Oil Red-O staining.

### Fluorescence immunostaining

Frozen sections were immune-stained with a vimentin antibody from Abcam (Cambridge, MA), and visualized with a Texas Red-conjugated secondary antibody. The nuclei were counterstained with 4’, 6-diamidino-2-phenylindole (DAPI). Images of each sample were acquired with fluorescence filters for Texas Red and DAPI. The two images from the same field were merged with NIS-Elements software if necessary.

### Statistical analysis

Results are presented as means ± SE (standard error). Differences in mean values were analyzed by a two-tailed t-test. A p value <0.05 and <0.01 were considered statistically significant and very significant, respectively.

## Results

### miR-144 targets vimentin in cultured cardiac endothelial cells

**Figure 1:**
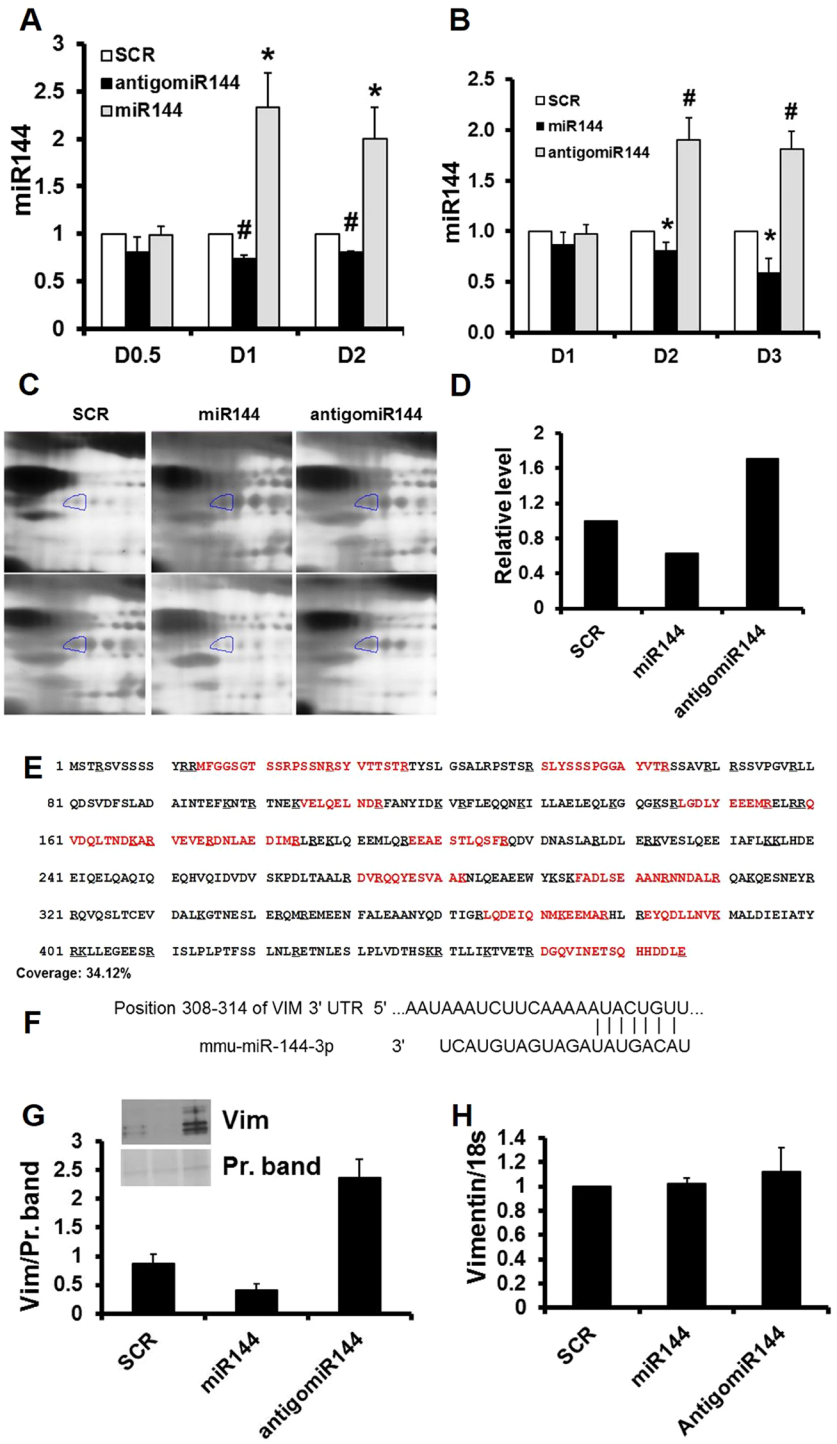
Vimentin is a miR-144 target in primary cultured cardiac endothelial cells. (**A**) Transfection. Cultured cardiac endothelial cells were transfected with miRNA and cellular miR-144 levels were determined by stem loop RT-PCR. N = 4, *p < 0.05 and ^#^p < 0.01 compared with SCR which is scrambled control. Dosage (D) 0.5 = 2.5 pmol, D1 = 5 pmol, and D2 = 25 pmol. (B**)** Incubation. Cultured cardiac endothelial cells were incubated with miRNA and cellular miR-144 levels were determined by stem loop RT-PCR. N = 4, *p < 0.05 and ^#^p < 0.01 compared with scrambled control. D1 = 5 pmol, D2 = 25 pmol, and D3 = 125 pmol. (**C**) Representative images of two-dimensional gel, n = 2. (**D**) Quantification of the circled two-dimensional gel spots from panel (**C**,**E)** Sequence coverage for mouse vimentin. Mass spectrometry detected peptides aligned with mouse vimentin sequences. The detected peptides are highlighted in red. (**F**) miR-144 targeting sequence in vimentin gene. (**G**) Western blot. Cultured cardiac endothelial cells were incubated with miRNA 144 and the culture medium was analyzed by Western blot for vimentin. N = 2. (**H)** Real time PCR. Cultured cardiac endothelial cells were incubated with miRNA 144 and the cells were harvested for quantification of total RNA. Vimentin mRNA was determined by real time PCR. N = 4. SCR: scrambled miRNA; Vim: vimentin.

To identify vascular targets, we manipulated miR-144 levels in primary cultured cardiac endothelial cells via transfection or incubation with synthetized miR-144. As shown in Fig. 1A,B, both incubation and transfection with miR-144 significantly increased intracellular miR-144, and antigomiR-144 decreased it. In initial experiments, transfection with Lipofectamine RNAiMAX led to increased cell death at higher concentrations, whereas incubation increased intracellular miR-144 levels to the similar degree, but without increasing cell death. Incubation was therefore chosen for subsequent experiments and reported below.

To identify proteins that resulted from the miRNA manipulation, the culture medium from miRNA treated endothelial cells was subjected to two-dimensional gel electrophoresis. After visualization with silver stain, 415 spots were reproducible detected across all the gels, and 5 affected spots were recovered for mass spectrum analysis. Nineteen proteins were identified from the 5 two-dimensional gel spots (supplementary table). One of these spots identified as vimentin was decreased by miR-144 but was increased in intensity by antigomiR-144 as shown in Fig. 1C,D. Seventeen vimentin peptide fragments were identified which covered 34.1% of the mouse vimentin primary sequence (Supplementary Table and Fig. 1E). Per web-based computer program search, a 7mer-8m miR-144 site at 308–314 of the mouse vimentin gene 3’ untranslated region was identified (Fig. 1F).

miR-144 suppressed but antigomiR-144 increased vimentin secretion in primary cultured endothelial cells, confirmed by Western blot (Fig. 1G). Vimentin mRNA was not affected by ether miR-144 or antigomiR-144 compared with scrambled control miRNA (Fig. 1H) indicating miR-144 suppression of vimentin is not mediated by mRNA degradation.

### **miR144 targets vimentin*in vivo***

**Figure 2:**
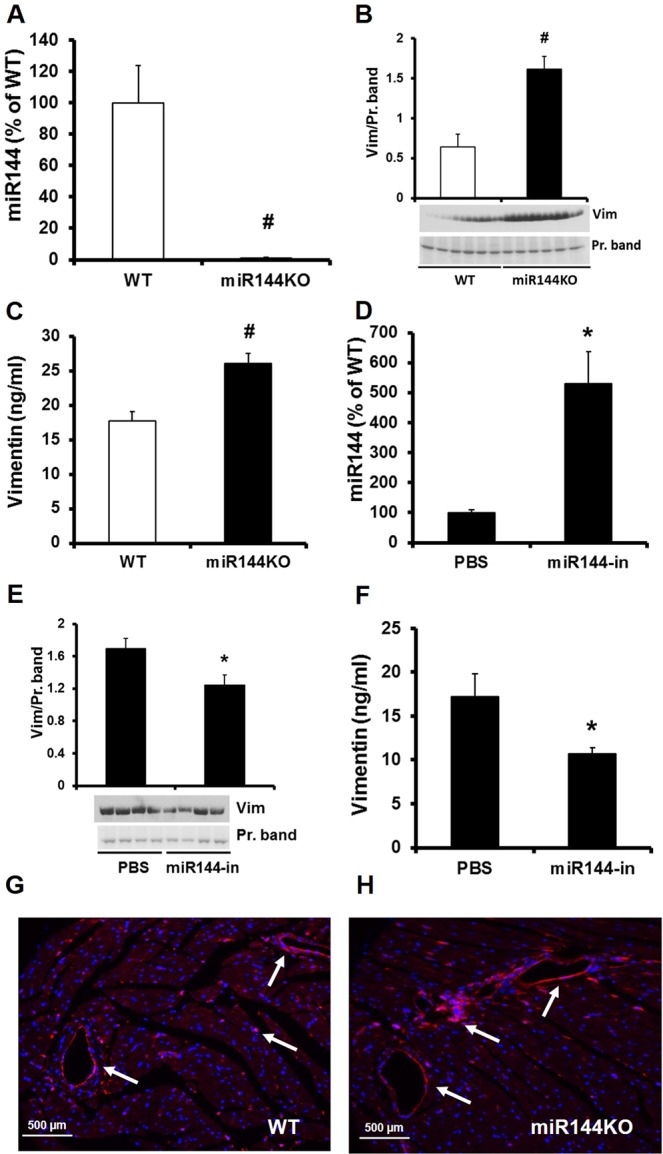
Confirmation of miR-144 targeting vimentin *in vivo*. miR-144 was knockdown in miR-144/451 KO mice (**A**) and vimentin levels in the plasma were determined by Western blot (**B**) and ELISA (**C**). N = 6 for both miR-144 KO mice and WT littermates. *p < 0.05 and ^#^p < 0.01 compared with WT littermates. miR-144 was raised by tail vein injection of miR-144 (**D**) and vimentin levels were determined by Western blot (**E**) and ELISA (**F**) assays from the mice after microRNA 144 injection. N = 4 for miR-144 injection and PBS control injection. *p < 0.05 compared with PBS injection. Vimentin was visualized by immunostaining (red color) in miR-144 KO (**H**) and WT littermate hearts (**G**). Each panel shows a representative pattern from KO (right panel) and WT mice (left panel). N = 6 for both WT and miR-144 KO. Vim: vimentin.

To confirm miR-144’s targeting of vimentin *in vivo*, we employed loss and gain function models e.g. ablation or injection of miR-144 in mice. Ablation of miR-144 decreased miR-144 by 98% (Fig. 2A) but very significantly increased circulating vimentin in the blood as evaluated by Western blot (Fig. 2B) and ELISA assay (Fig. 2C). Tail vein injection of synthetized miR-144 increased miR-144 by 531% (Fig. 2D) and significantly decreased circulating vimentin (Fig. 2E,F).

To better understand vimentin localization in the cardiovascular system, we performed immunostaining on frozen sectioned heart tissue. As shown in Fig. 2G,H, though ubiquitous, vimentin was predominantly expressed in the blood vessels.

### **Ablation of miR144 promotes atherosclerotic plaque formation, leads to fatty liver, and modifies cardiac structure**

Vimentin has been detected in the human coronary atherosclerotic plaque^17^. It is also known that vimentin is involved in platelet adhesion leading to thrombosis^6^ and so may potentially play a role in early atherogenesis. We therefore investigated if miR-144 KO mice, which displayed increased circulating vimentin, are more susceptible to atherosclerosis. miR-144 KO mice were fed HFD for 48 weeks. These KO mice consumed similar amounts of chow compared with WT littermates and had a similar growth curve (supplementary Fig. 1A,B).

**Figure 3:**
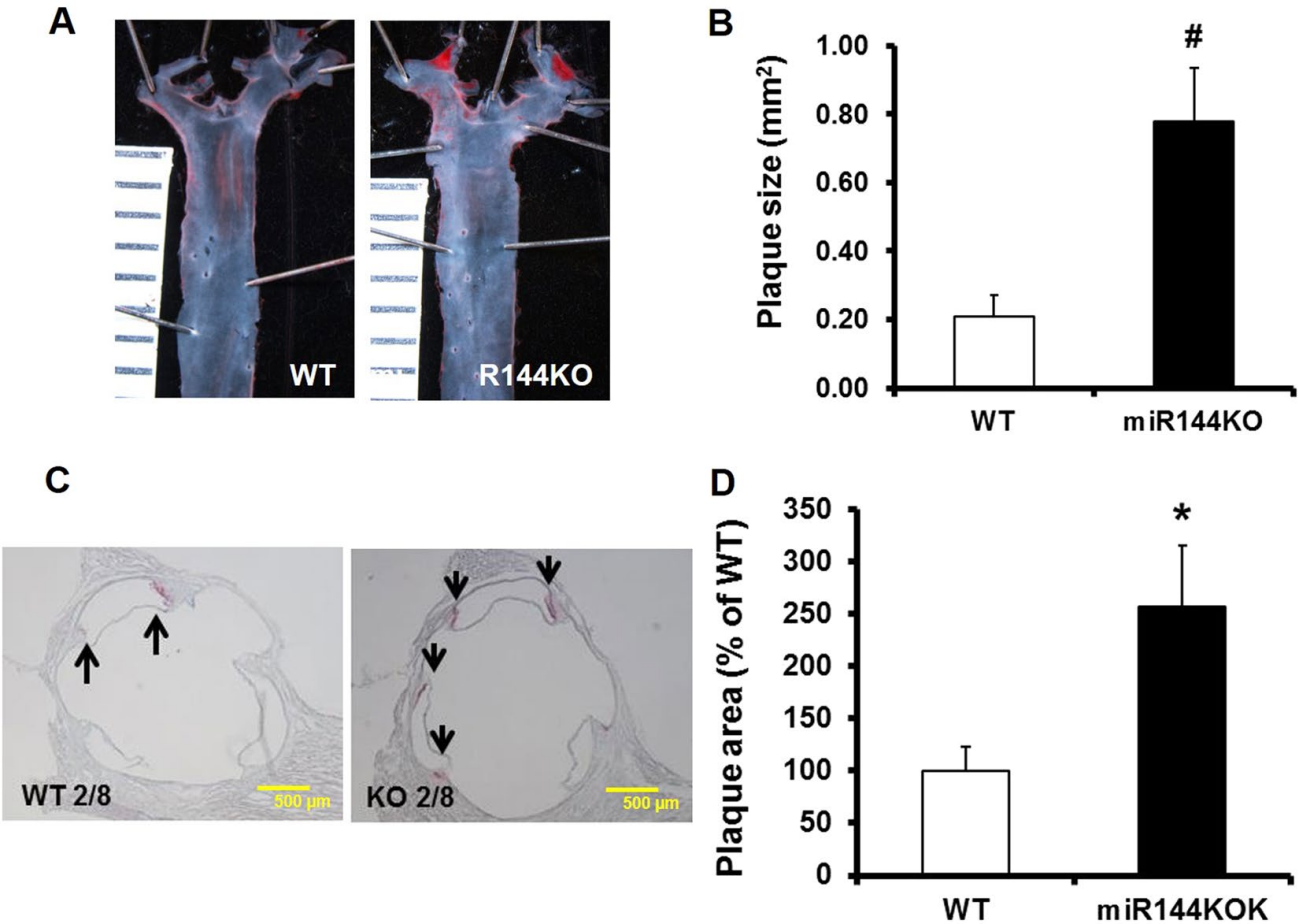
Ablation of miR-144 promotes atherosclerosis *in vivo*. miR-144 KO mice and WT littermates were fed HFD. Atherosclerotic plaques were evaluated in aortic tree (**A**) and root area (**C**) after Oil Red-O stain into red color. Atherosclerotic plaques in the aortic tree and root areas were quantified with Image J and summarized in panel (**B**,**D)** respectively. N = 10 for miR-144 KO mice and WT littermates. *p < 0.05 and ^#^p < 0.01 compared with WT littermates. KO: miR-144 KO. Scale in panel A was 1 mm.

We next searched for atherosclerotic plaques in the aorta. Atherosclerotic plaques were only found in aortic root and arch areas at bifurcation points. Oil Red-O stained en-face views of the aortic tree showed that miR-144 KO mice formed plaques that were approximately 4 times larger than WT littermates (Fig. 3A,B). Furthermore, Oil Red-O stained continuous sections of aortic root area showed that miR-144 KO mice formed more atherosclerotic lesions (Fig. 3C,D), with most plaques forming in the junction area (commissure) between the interior wall of the aorta and semilunar valve leaflets (supplementary Fig. 2).

**Figure 4:**
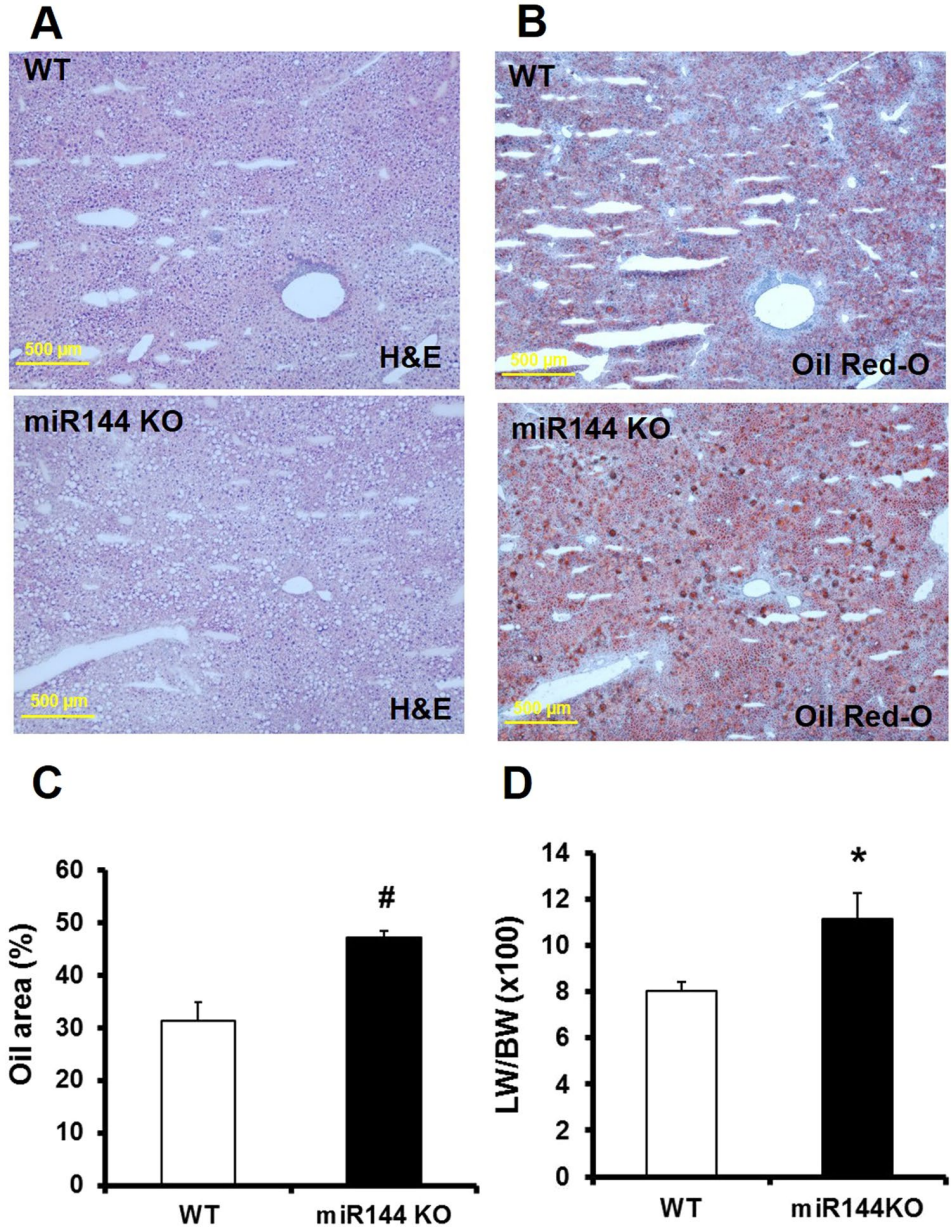
Ablation of miR-144 increases fatty liver disease. Lipid droplets were visualized by hematoxylin and eosin (**A**) and Oil Red-O stain (**B**). (**C)** Quantification of the stained oil droplets from panel B. N = 10 for miR-144 KO mice and WT littermates, ^#^p < 0.01 compared with WT littermates. (**D)** Liver weight. N = 10 for miR-144 KO mice and littermates, *p < 0.05 compared with WT littermates. H&E: Hematoxylin and eosin stain. Scale bar in panel A and C: 500 µm.

Given prior reports, we expected that miR-144 KO mice would display more severe fatty liver disease compared with WT littermates. Histological stained liver sections show that miR-144 KO resulted in more lipid droplets as shown in Fig. 4A (round dots, hematoxylin and eosin stain) and Fig. 4B (red dots, Oil Red-O stain). The lipid droplets occupied much more space in miR-144 KO liver than that in WT littermates (Fig. 4C). The liver weight was also much higher than WT littermates as measured by liver weight and body weight ratio (Fig. 4D). Liver function assessed from levels of aspartate aminotransferase and alanine aminotransferase trended to be higher in miR-144 KO mice compared with WT littermates, but there is statistically no difference (Supplementary Fig. 3A,B).

The heart weight was also increased in miR-144 KO mice compared with WT littermates (Supplementary Fig. 4A), but left ventricular function (as indicated by ejection fraction from echocardiography) was no different between miR-144 KO and WT littermates (Supplementary Fig. 4B,C). Unexpectedly, the hearts were characterized by gross dilatation of the right ventricle in the miR-144 KO mice (Supplementary Fig. 4D), which may have contributed to the increased heart weight, an unexpected and novel finding which remains unexplained, given that hemodynamic assessment of the mice was not within the design of this study.

### Circulating cholesterol is not modified by miR-144 KO

**Figure 5:**
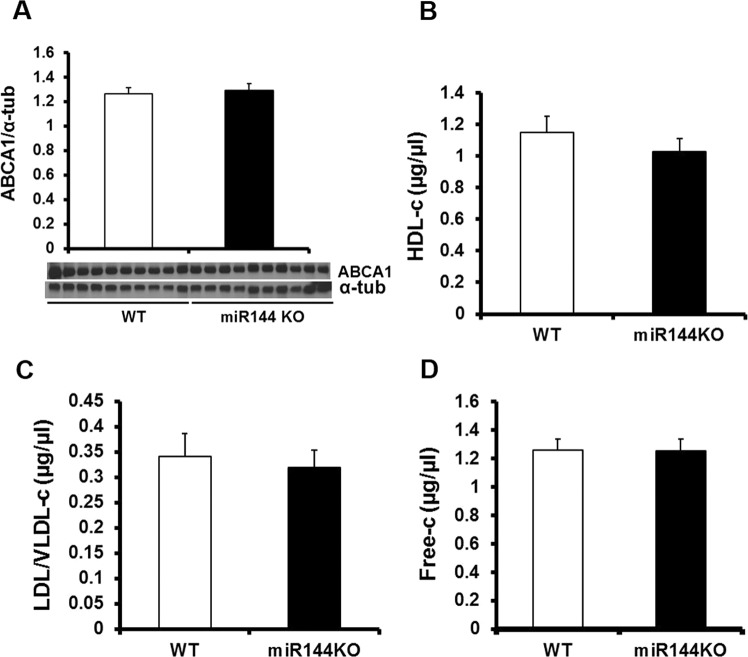
ABCA1 and cholesterol homeostasis were not affected by miR-144 KO. A. Western blot. Tissue extracts from liver were subjected to Western blot. N = 10 for both miR-144 KO mice and WT littermates. Plasma cholesterol including high density lipoprotein cholesterol (**B**), LDL/VLDL cholesterol (**C**), and free cholesterol (**D**) were determined with a commercial kit as described in Materials and Method. N = 10 for miR-144 KO mice and WT littermates.

Previous studies have reported that miR-144 regulates cholesterol efflux via targeting ATP binding cassette transporter A1 (ABCA1)^9,10^ and agomiR-144 has accelerated plaque formation in apolipoprotein E (ApoE) KO mice^11^. Because of this, and our findings of increased atherosclerotic burden in miR-144 KO’s, we next measured ABCA1 and cholesterol levels in HFD fed miR-144 KO mice. As shown in Fig. 5A, liver ABCA1 protein levels were similar in miR-144 KO mice and WT littermates. Similarly, the circulating cholesterol levels were no different between miR-144 KO mice and WT littermates in terms of high-density lipoprotein cholesterol, LDL/VLDL cholesterol, or free cholesterol (Fig. 5B–D). We conclude that ablation of miR-144 promotes plaque formation in miR-144 KO mice, and is not mediated by changes in ABCA1 or cholesterol homeostasis.

### Aortic vimentin accumulation may contribute to atherosclerotic plaque formation

**Figure 6:**
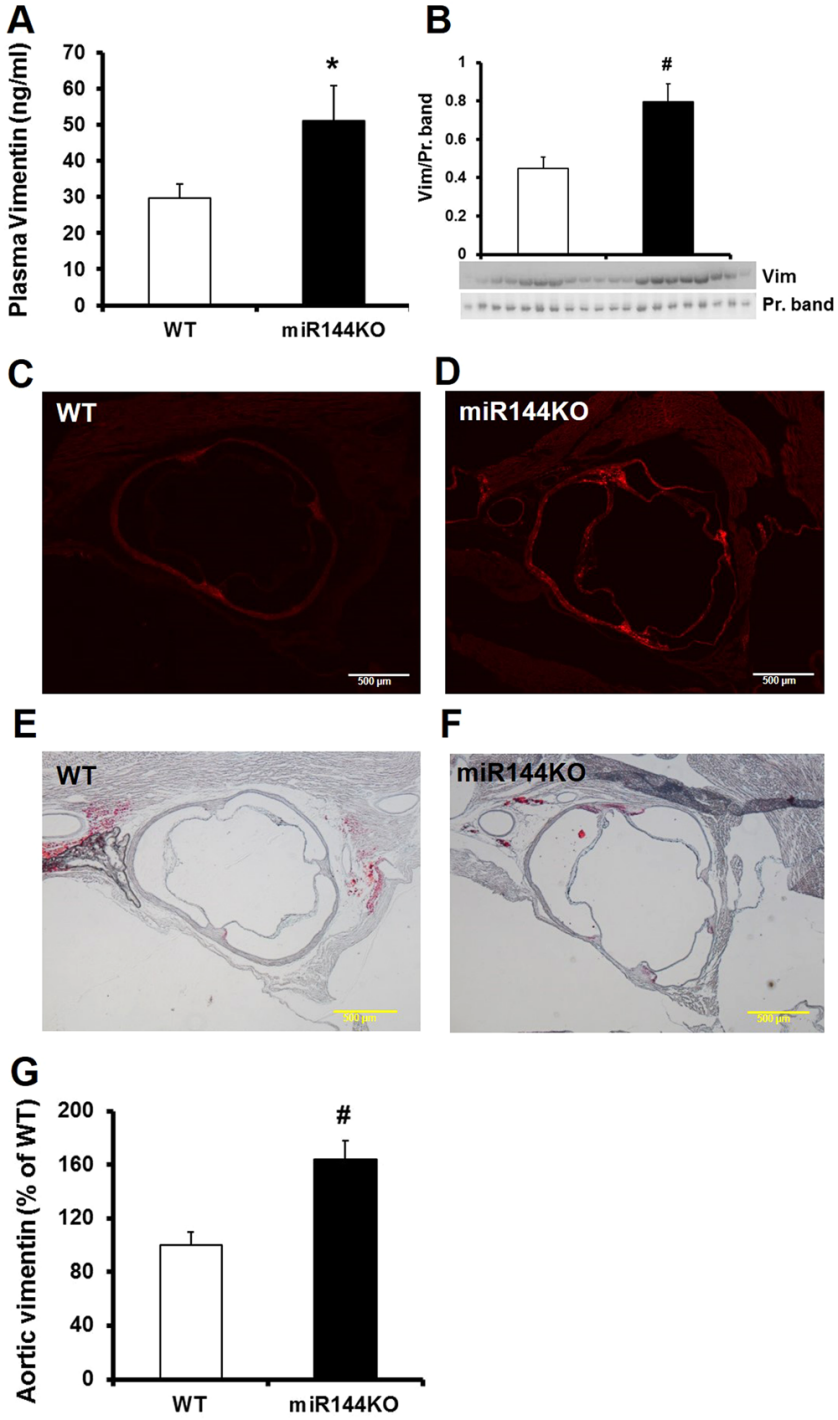
Elevation of vimentin in miR-144 KO mice is collocated with atherosclerotic plaques. Plasma vimentin was determined by ELISA (**A**) and Western blot (**B**). N = 10 for miR-144 KO mice and WT littermates, *p < 0.05 compared with WT littermates. Vimentin in the aortic root was visualized by immunostaining (**C,D**) in comparison with that adjacent tissue cuts stained with Oil Red-O (**E,F**). Each panel of picture is representative of data from 10 mice. The vimentin stains were quantified by Image J and summarized in panel (**G**) N = 10 for both miR-144 KO mice and WT littermates, ^#^p < 0.01 compared with WT littermates. Scale bar in panel C, D, E, and F: 500 µm. Vim: vimentin.

We finally investigated vimentin concentration and its distribution in the HFD-fed miR-144 KO mice. Circulating vimentin levels were significantly higher in the miR-144 KO mice as determined by ELISA assay and Western blot analysis (Fig. 6A,B). Immunostaining of the aortic root section showed that vimentin was predominantly expressed in the aortic root and the intensity of vimentin staining was much higher in the miR-144 KO aortic root than that in the WT littermates (Fig. 6C,D,G). It was most densely expressed in the commissural area which is particularly susceptible to atherosclerotic plaque formation. Side by side comparison of staining with Oil Red-O and vimentin showed that vimentin and atherosclerotic plaque are highly associated (Fig. 6C–F). We postulate that the accumulated vimentin in the aorta promotes atherosclerotic plaque formation in the miR-144 KO mice.

## Discussion

In this study we identified, using proteomic analysis and subsequent *in-vitro* and *in-vivo* models, that miR-144 targets vimentin which, in turn, is associated with enhanced atherogenesis in miR-144 KO mice. Ablation of miR-144 increased circulating and tissue (predominantly in the vascular wall) vimentin and intravenous miR-144 injection decreased it. Furthermore, ablation of miR-144/451 promoted atherosclerotic plaque formation in the area of aortic root and more distal aorta, was associated with fatty liver, and unexpectantly was characterized by gross right ventricular dilation, but normal left ventricular function.

Contrary to previous reports in other mouse models^9,10^, increased atherogenesis could not be explained by alterations of cholesterol efflux via targeting ABCA1 expression, or circulating cholesterol levels in our miR-144 KO mice fed HFD. However, in our distribution studies we demonstrated for the first time that vimentin is accumulated in the commissural area of aortic root which is highly susceptible for atherosclerotic plaque formation. Given the known functions of vimentin, and the association of vimentin and atherosclerotic plaques, our data suggests that miR-144 by its effects on vimentin, may play a role in atherogenesis.

Using TargetScan (http://www.targetscan.org/vert_72/), we identified 760 transcripts containing miR-144 sites. The functional importance of some of these 760 transcripts has been confirmed in different research areas and different model systems such as ABCA1 in atherosclerosis^11^, Rac1 in ischemic preconditioning-mediated cardioprotection^18^, autophagy signaling in left ventricular remodeling after myocardial infarction^19^, Zeb1/Lox in extracellular matrix remodeling after myocardial infarction^16^, and EGFR (epidermal growth factor receptor) in inhibition of liver tumor growth^12^. Though not reported, we identified a 7mer-8m miR-144 site at 308–314 of the mouse vimentin gene 3’ untranslated region. We are thus the first to report that vimentin is a target of miR-144 and have confirmed a functional relationship with atherosclerosis in miR-144 KO mice.

Vimentin acts primarily as an intermediate filament protein in the cytoskeleton, but is also secreted into blood by active macrophages and other source cells^20,21^. Vimentin also interacts with other macromolecules implicated in atherosclerosis including oxysterol-binding protein related protein after binding 25-hydroxycholesterol^22^, and human arachidonate 12/15-lipoxygenase promotor as a promoter binding protein leading to alteration of lipoxygenase activity^23^. Vimentin deficiency in macrophages has been shown to attenuate atherosclerosis in mice^24^. It has also been detected in the necrotic core and areas of active inflammation of atherosclerotic lesions^17^. Our data showed that ablation of miR-144 increased plasma vimentin (Fig. 2B,C) and tail vein-injection of synthesized miR-144 decreased it (Fig. 2E,F). Immunostaining showed that the increased vimentin in miR-144 KO heart was predominantly present in the vasculature (Fig. 2G,H). This finding prompted us investigate how increased vimentin might affect atherogenesis in miR-144 KO mice as it is known that vimentin is involved in platelet adhesion^6^ and the initiation atherosclerosis via interactions with VWF^5,17,25^. Accumulating evidence suggests that vimentin plays a key role in cell adhesion via interacting with endothelial cell surface proteins such as VWF, a large plasma adhesive glycoprotein with mutimeric structure. VWF traps platelets in atherosclerosis-prone sites via binding to its platelet receptor glycoprotein Ib domain, and VWF deficient mice have reduced atherosclerosis^26^. Examining the relationship between miR-144-vimentin signaling and subsequent early stages of atherogenesis via WVF-induced endothelial-platelet interactions warrant further investigation.

The impact of altered vimentin signaling may go beyond its putative role in early atherogenesis via these endothelial-platelet interactions. Vimentin is a marker of epithelial-to-mesenchymal transition, a critical event in induction of cell motility involved in multiple biological processes including wound healing, cancer metastasis, and embryonic development^27^. Vimentin is also a prerequisite for epithelial-to-mesenchymal transition and positively regulates epithelial-to-mesenchymal transition^28^. Endothelial to mesenchymal transition is also common in maturing atherosclerotic lesions^29^. Consistent with previous reports that the surface vimentin is increased in atherosclerotic lesion compared with that in nonlesion vasculature^30^, we observed significantly increased vimentin levels in miR-144 KO aortic root compared with WT littermates which is consistent with atherosclerotic lesions (Figs. 6C–F, 3C,D). Future studies should examine the possible role of increased vimentin in terms of cellular transformation within the lesions.

Finally, and although not a primary focus of our study, miR-144 KO fed HFD was associated with fatty liver disease. Nonalcoholic fatty liver disease is an independent risk factor for atherosclerosis^31^. Our data showed that miR-144 KO resulted in massive fat accumulation in the liver and increased liver weight (Fig. 4D). Many mechanisms of nonalcoholic fatty liver disease leading to atherosclerosis have been postulated including dyslipidemia, inflammation, oxidative stress, and etc^32^. Alteration of cholesterol metabolism, at least in terms of ABCA1 regulation, and circulating cholesterol levels cannot explain increased atherosclerosis in our animals. This is despite previous reports suggesting miR-144 decreases high density lipoprotein cholesterol via targeting liver and macrophage ABCA1^9,10^ and another study showing agomiR-144 accelerates plaque formation in ApoE KO mice^11^. Our data showed that ablation of miR-144 had no effect on liver ABCA1 or plasma levels of cholesterol, but still promoted atherosclerotic plaque formation. This discrepancy in effects of miR-144 on atherosclerosis requires further study, but may stem from (1) the animal used in these studies, miR-144 KO mice, since previous studies showed that ablation of genes at different developmental stages may result in different phenotypes^27^; or (2) the very prolonged HFD protocol (48-weeks) that we used, which may have different effects compared with shorter term protocols^33^.

In conclusion, we identified vimentin as a miR-144 target and describe its potential association with increased atherosclerosis in miR-144 KO mice fed an HFD. These novel findings provide the basis for future studies of the role of miR-144 in human atherogenesis.

## Data Availability

All data generated or analyzed during this study are included in this published article and Supplementary Material.

## Supporting information

Supporting information.
