# Supplementary material for: Ablation of miR-144 increases vimentin expression and atherosclerotic plaque formation
Source: Sci Rep. 2020 Apr 9;10:6127. doi: 10.1038/s41598-020-63335-7 (PMC7145828; doi:10.1038/s41598-020-63335-7)
Supplement: Supplementary file 1 — Supporting information. [file 41598_2020_63335_MOESM1_ESM.docx]

**Supplemental Material**

**Ablation of miR-144 increases vimentin expression and atherosclerotic plaque formation**

Quan He^1^, Fangfei Wang^1^, Takashi Honda^1^, Kenneth D. Greis^2^, and Andrew N. Redington^1^*

^1^The Heart Institute, Cincinnati Children’s Hospital Medical Center, Cincinnati, Ohio, United States of America

^2^College of Medicine, University of Cincinnati, Cincinnati, Ohio, United States of America

*Corresponding author

[Andrew.redington@cchmc.org](mailto:Andrew.redington@cchmc.org) (AR)

**sTable. Mass spectrometry identified mouse proteins.** Cardiac endothelial cells were treated with miR-144 miRNAs and the conditioned culture medium was subjected to two-dimensional gel analysis. The intensity of the two-dimensional gel spots which was decreased by miR-144 and increased by antagomiR-144 were recovered and subjected to mass spectrometry. Nineteen proteins were identified from 5 two-dimensional gel spots.

**
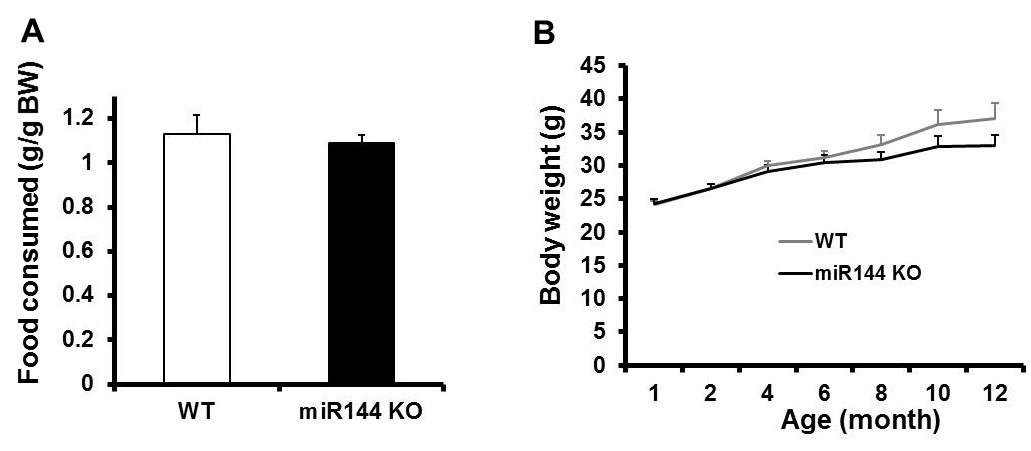
**

**sFigure 1. Physiological parameters of HFD-fed mice.** miR-144 KO mice and WT littermates consumed similar food in a two-week monitoring period (**A**) and their body weight gain was similar during 12 month period (**B**). N = 10 for both miR-144 KO mice and WT littermates.


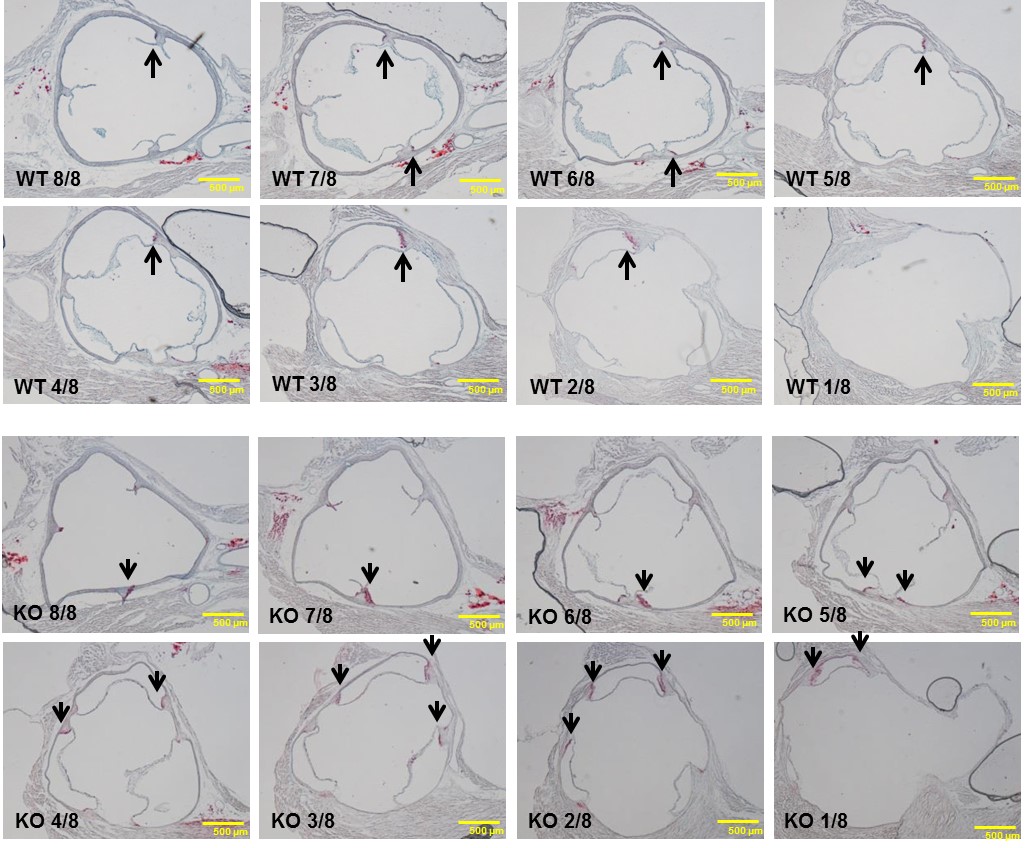


**sFigure 2. Represent Oil Red-O stains of an entire set of serial sections.** The aortic root was continuously sliced and stained with Oil Red-O. Each set of aortic root sections was composed of 8 slides. Quantification of plaque area presented in Fig. 3D was based on the whole set slides of aortic roots. Atherosclerotic plaques illustrated by arrows. WT: wild type; KO: miR144 knockout.


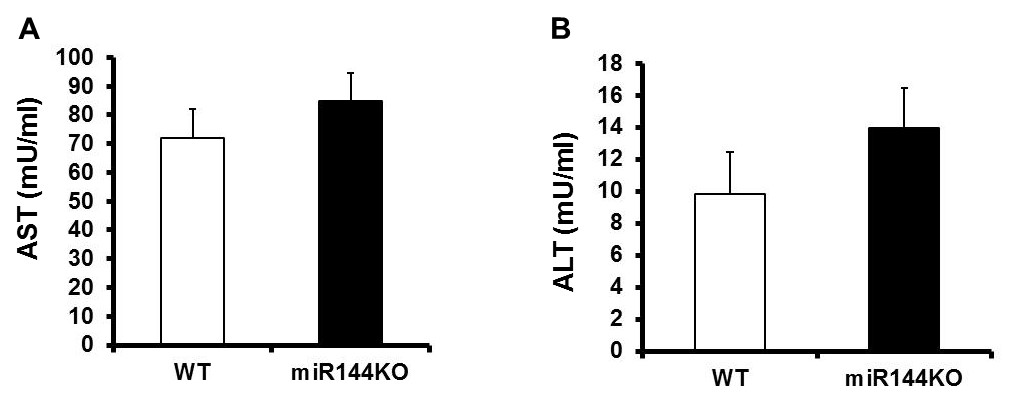


**sFigure 3.** **Liver function testing.** Aspartate aminotransferase (AST, **A**) and alanine aminotransferase (ALT, **B**) were assayed with kits from BioVision. Both AST and ALT tend to increase in miR-144 KO mice under HFD challenge compared with WT littermates but did not reach statistically difference. N = 10 for both miR-144 KO mice and WT littermates.


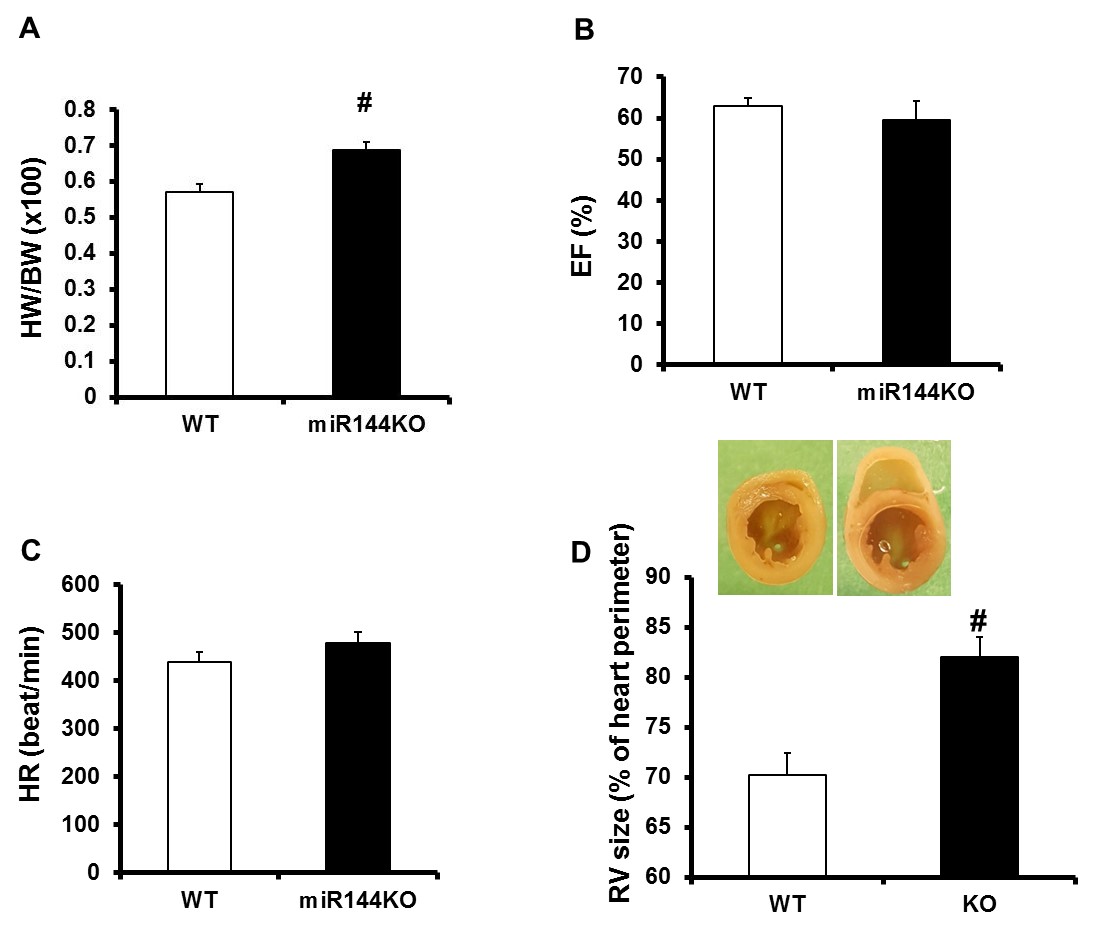


**sFigure 4. Cardiac characteristics of miR-144 KO mice.** Heart weight and body weight ratio (HW/BW) was increased in miR-144 KO mice compared with WT littermates (**A**). This was explained by right ventricular dilatation in miR-144 KO mice (**D**). Cardiac function represented by ejection fraction (EF, **B**) and heart rate (HR, **C**) was no different between miR-144 KO mice and WT littermates. *p < 0.05 and ^#^p < 0.01 compared with WT. N = 10 for both miR-144 KO mice and WT littermates.

Protein stain


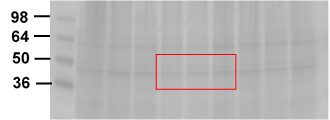


Western blot


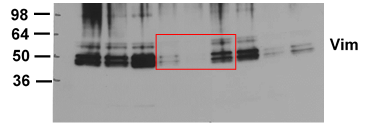


**sFigure 5. Full-length blots for Fig. 1G.** The blot was stained with MemCode and documented. The blot was subjected to western blot after the MemCode stain was removed.

Protein stain


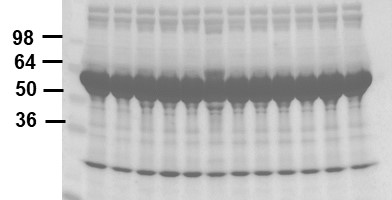


Western blot


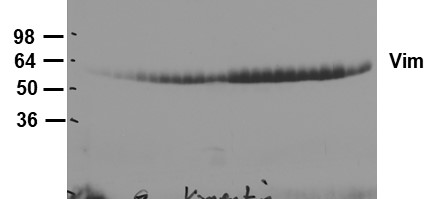


**sFigure 6. Full-length blots for Fig. 2B.** The blot was stained with MemCode and documented. The blot was subjected to western blot after the MemCode stain was removed.

Protein stain


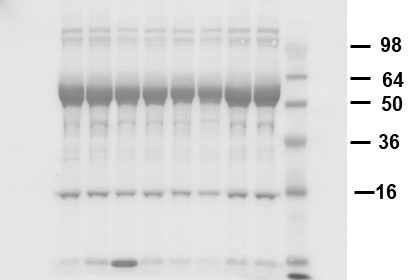


Western blot


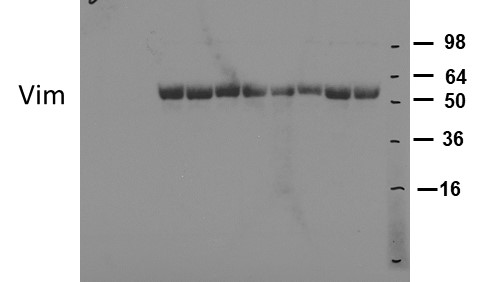


**sFigure 7. Full-length blots for Fig. 2E.** The blot was stained with MemCode and documented. The blot was subjected to western blot after the MemCode stain was removed.


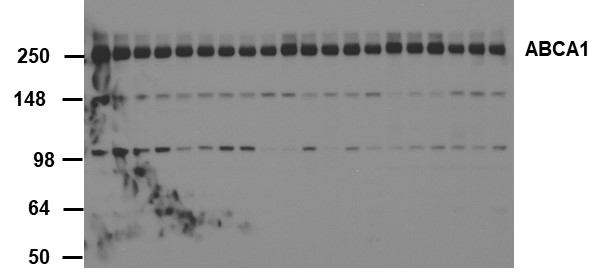


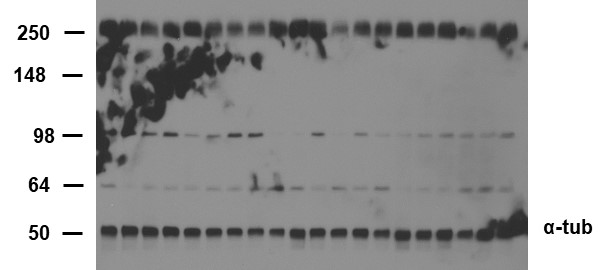


**sFigure 8. Full-length blots for Fig. 5A.** The blot was first probed with ABCA1 antibody and striped/re-probed with α-tubulin antibody.

Protein stain


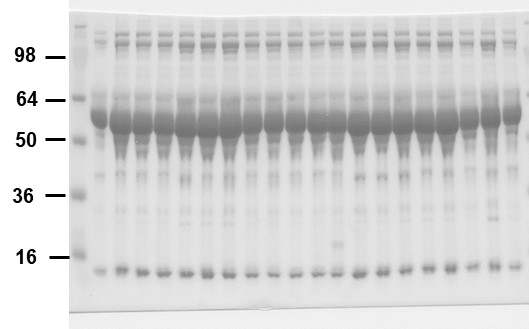


Western blot


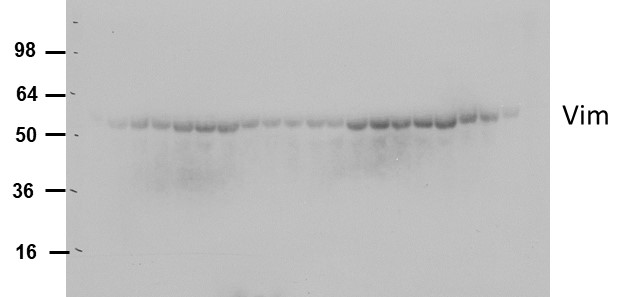


**sFigure 9. Full-length blots for Fig. 6B.** The blot was stained with MemCode and documented. The blot was subjected to western blot after the MemCode stain was removed.
